# Influence of Reference Gene Selection on miRNA Quantification by RT-qPCR in Human Placental Samples

**DOI:** 10.3389/bjbs.2025.15354

**Published:** 2025-12-04

**Authors:** Ankica Sekovanić, Tatjana Orct, Adrijana Dorotić, Daria Pašalić, Zorana Kljaković-Gašpić, Sandra Stasenko, Tatjana Mioč, Martina Piasek, Jasna Jurasović

**Affiliations:** 1 Institute for Medical Research and Occupational Health, Zagreb, Croatia; 2 University Hospital Sveti Duh, Zagreb, Croatia; 3 University of Zagreb, School of Medicine, Zagreb, Croatia; 4 Merkur University Hospital, Zagreb, Croatia; 5 Poliklinika Harni, Zagreb, Croatia

**Keywords:** MicroRNA, normalization, housekeeping genes, RT-PCR, placenta

## Abstract

The gold standard for assessing expression of miRNAs, small molecules involved in numerous biological processes, is reverse transcription-quantitative polymerase chain reaction (RT-qPCR). The reliability of RT-qPCR analysis results largely depends on accurate data normalization and the selection of an appropriate reference gene. This study evaluated the stability of five candidate reference genes—miR-525, miR-520c, SNORD48, miR-135b, and miR-143—in human placental samples. GeNorm, NormFinder, BestKeeper, and the delta Ct-method were used to evaluate gene expression stability. The effect of reference gene selection for normalization of target miRNAs (miR-1537, miR-190b, miR-16, miR-21, and miR-146a) expression in term placental samples from smokers and non-smokers was also investigated. All statistical tools identified miR-525, miR-520c, and SNORD48 as the three most stable reference genes, except for GeNorm, which recommends the combination of the first two genes. Normalization using SNORD48 and miR-525 produced comparable results for miR-21 expression in the placental samples, both in smokers and non-smokers, whereas normalization with miR-143 yielded markedly different outcomes compared to SNORD48 and miR-525. These findings highlight the considerable impact of reference gene selection on RT-qPCR results, emphasizing the importance of careful validation to avoid misinterpretation of gene expression data.

## Introduction

MicroRNAs (miRNAs) are small (18–20 nucleotides) non-coding endogenous RNA molecules that play crucial roles in numerous biological processes, including cell proliferation and differentiation [1]. The gold standard for detection of miRNA expression is reverse transcription-quantitative polymerase chain reaction (RT-qPCR). The cycle threshold (Ct), a key parameter obtained from RT-qPCR, is the number of cycles that the samples experienced before detection. The most commonly used method for analysing RT-qPCR data is relative quantification, which involves comparing the PCR signal of a target gene to that of a reference (or normalizer) gene, recorded as ΔCt [2, 3]. This approach helps reduce discrepancies arising from sample quality or processing variations. Although guidelines for the standardization of RT-qPCR analysis recommend using validated reference genes for normalization [4], there is no universally accepted reference gene (“housekeeping” gene) for normalizing RT-qPCR data. According to Schwarzenbach et al. [5], a good reference gene/genes should exhibit stable expression with minimal standard deviation, as well as high extraction specificity, indicating that expression and extraction specificity are not significantly influenced by sample storage or processing conditions. Furthermore, relative normalization approach of RT-qPCR data using reference genes is better than the absolute normalization approach, which calculates miRNA expression using standard curves derived from synthetic miRNAs and melting curves [5]. To date, only one study has specifically evaluated Ct and ΔCt values obtained with and without a reference gene–18S rRNA (ribosomal RNA)–for miR-371a-3p, miR-372, and miR-373-3p in the serum of cancer patients [6]. Although their findings revealed a strong correlation between Ct and ΔCt values (R^2^ = 0.956), indicating that RT-qPCR can be used without endogenous control in their specific case, Wang et al. [7] emphasized the necessity of reference-gene-based normalization for accurate RT-qPCR data interpretation. Choosing an appropriate reference gene is essential to prevent data misinterpretation and to identify actual shifts in miRNA expression levels. The most common approach for selecting a reference gene is based on literature data, manufacturer recommendations, and low standard deviation values in miRNA microarrays. Small nuclear/nucleolar RNAs are the most frequently employed as normalizers due to their stability and good expression. However, their expression levels can be affected by various diseases and/or experimental conditions, making it important to ensure that the selected normalizer is suitable for the analysed samples [5, 8, 9]. As we mentioned earlier, normalization is a crucial step in RT-qPCR analysis because tissue-specific expression patterns and cellular composition can vary substantially. Consequently, reference genes verified in one tissue type might not be suitable for another. Human placenta is a unique and heterogeneous organ with dynamic changes in gene expression throughout gestation, making accurate normalization particularly challenging. The literature shows a lack of studies that assess potential reference miRNAs in placental tissue. Therefore, the goal of the current study was to identify reliable reference genes for measuring miRNA in the human placenta. Candidate reference genes were selected based on manufacturer recommendations, prior reports of miRNAs with high expression in placental tissue [10, 11] and/or their use as housekeeping genes in previous studies [12]. We examined the stability of five candidate reference genes in human placental samples using different statistical tools, namely GeNorm, NormFinder, BestKeeper, and the delta Ct-method. Additionally, we investigated the impact of different normalization genes on the quantification of the relative expression of five target miRNAs (miR-1537, miR-190b, miR-146a, miR-16, miR-21) in human term placental tissue samples, each previously linked to maternal smoking and/or exposure to toxic metals (Cd and Pb) [13, 14].

## Materials and Methods

### Sample Collection

The term placental samples (n = 64) for this methodological study were collected between 2018 and 2019 at the Merkur University Hospital’s maternity ward in Zagreb, Croatia, as part of a larger research project (METALORIGINS, HRZZ-IP-2016-06-1998). The project protocols were reviewed and approved by the ethical committees of the participating institutions, ensuring compliance with the principles of the Helsinki Declaration. All study participants completed written informed consent form before sample collection. Immediately after delivery, placental samples were placed in sterile zip-lock polyethylene bags and transported to the analytical laboratory within 2 hours. Upon arrival, the remaining umbilical cord was removed and the placental membranes were trimmed. To ensure representative samples among participants, full-thickness samples were cut using a titanium or ceramic knife from the central part of the placental disc, avoiding the site of umbilical cord insertion. The 1–2 mm of decidua basalis and chorionic plate were carefully dissected with a knife to obtain trophoblastic tissue [15, 16] and stored at −80 °C until miRNA isolation.

### Real-Time PCR Quantification (RT-qPCR)

miRNAs were isolated from placental samples (average weight 52 mg; range 41–67 mg) using the miRNeasy Mini Kit (Qiagen, Hilden, Germany) following the manufacturer’s protocol. Frozen placental samples were homogenized in three 15 s bursts at 7800 rpm in QIAzol Lysis Reagent using a Combo Precellys Evolution (Bertin Technologies, Montigny-le-Bretonneux, France) to ensure complete homogenization. A NanoPhotometer P360 (Implen, München, Germany) was used to measure the quality and concentration of isolated mRNA enriched with miRNAs, and RNA integrity was evaluated by measuring absorbance at 260/280 (acceptable range 1.9–2.1). Reverse transcription into cDNA was made immediately after isolation using a Qiagen miScript II RT Kit in a 20 µL reaction volume with 2 µL of isolated miRNAs following the manufacturer’s protocol. The reaction was incubated on a ThermoMixer C (Eppendorf, Hamburg, Germany) at 37 °C for 1 h, and 5 min at 95 °C, and cDNAs were stored at −20 °C until further analysis. The expression of miRNAs (miR-1537-3p, miR-190b, miR-16-5p, miR-21-5p, and miR-146a-5p, miR-525-5p, miR-520c-3p, SNORD48, miR-135b-5p, and miR-143-3p) was quantified by RT-qPCR using a custom miScript miRNA PCR array with 96 wells and a miScript SYBR Green PCR Kit (Qiagen, Hilden, Germany) according to the manufacturer’s protocol. miScript PCR Array contained miRNA-specific miRNA Primer Assay, while SYBR Green Kit contained the miScript Universal Primer (reverse primer). The Custom miScript miRNA PCR arrays were designed using the Qiagen GeneGlobe online platform, which enables the selection of particular primers of miRNAs of interest from the commercially available primer set. The cDNA obtained from placenta samples was diluted (1:10) before RT-qPCR analysis, following the manufacturer’s protocol used in the study to reach the recommended range of total amount per sample. The goal of this dilution was to reduce potential inhibitory effects from the sample matrix while maintaining enough template for adequate amplification. The RT-qPCR analysis was conducted on an AB7500 (Applied Biosystems, Waltham, Massachusetts, USA) under the following conditions: an initial activation step at 95 °C for 15 min, followed by three-step cycling (denaturation at 94 °C for 15 s, annealing at 55 °C for 30 s, and extension at 70 °C for 30 s) in a total of 40 cycles. Each RT-qPCR plate included a miRNA reverse transcription control (miRTC) and a positive PCR control (PPC) to verify the technical reproducibility of reverse transcription (RT) and PCR amplification, respectively, also permitting comparison across plates. In accordance with the manufacturer’s protocol, a ΔCt value (Ct_miRTC_–Ct_PPC_) < 7 indicated no inhibition of the RT reaction is apparent, while values ≥ 7 is evidence of impurities that may have inhibited the RT reaction and required repetition of the entire workflow, including isolation. After RT-qPCR, amplification signals were computed with 7500 software v2.0.6 (Applied Biosystems), and the cycle threshold (Ct) values were determined accordingly.

### Statistical Analysis

Statistical analysis was performed using R software package, version 4.2.2 (R Foundation for Statistical Computing, Vienna, Austria), and TIBCO Statistica™, version 14.0.0.15 (TIBCO Software, Inc., Palo Alto, CA, USA). An *a priori* power analysis was conducted in G*Power (version 3.1.9.4; Heinrich Heine University Düsseldorf, Düsseldorf, Germany) to determine the minimum sample size required to detect a statistically significant difference between analysed groups, assuming adequate study power. The estimate was based on literature data [13] and preliminary results for candidate miRNAs in the placenta of smokers and non-smokers, with a study power of 80%. Power analysis indicated that at least 22 samples per group were required to detect differences between-group (smokers vs. non-smokers) in placental miR1537 expression, whereas smaller group sizes were sufficient for the other miRNAs. For miR-1537, when amplification was not detected or when Ct values exceeded 35.4 (about 25% of samples), we used an imputation method, assigning a value of 36, which represents the critical number of cycles plus one (Ct +1) [17, 18]. This approach ensured that all biological replicates were retained, preventing the loss of statistical power, and minimizing bias that could arise from listwise exclusion of such samples. The Ct values for all other miRNAs were below 35.4 and required no imputation. The coefficient of variation (CV), as a measure of precision, was calculated as the standard deviation divided by the mean of a group of replicates. The BestKeeper Index (BKI) was calculated as the z-root of the product of all reference genes Ct values–Ct_i_, where *i* represents each reference gene and z denotes the total number of reference genes included in the calculation [19]. Pearson’s correlation coefficients (r) were used to evaluate inter-gene relations among all reference genes, as well as between reference genes and BKI. For GeNorm and NormFinder methods, Ct values were converted to relative quantities using the comparative Ct method (2^(−ΔCt)^), whereas ΔCt was calculated using equation: ΔCt = Ct _(i)_ – Ct _(highest expressed)_. The log_2_-transformed expression ratio (a_ij_/a_ik_) of each pairwise reference gene combination (j, k) was calculated to obtain the normalization factor. The least stable reference gene was systematically excluded during the stepwise process until the most stable pair of reference genes was identified. Pairwise variation was calculated for each reference gene relative to all other reference genes. Gene stability in GeNorm was assessed using the M-value, defined as the average of pairwise variation [20], while in NormFinder, gene stability was calculated as the ratio of intra-group variation to overall expression variation of the candidate gene [21]. In delta Ct method, the optimal combination of reference genes was assessed based on the variability of their Ct values across samples. Gene stability was calculated as the average standard deviation obtained from pairwise comparisons with other reference genes [22]. The ΔCt values were computed using the equation: ΔCt = Ct-miRNA–Ct-normalizer, where miR-525, miR-520c, SNORD48, miR-135b, and miR-143 were used as normalizers for placental samples. Relative expression levels of miRNAs of interest were calculated using the 2^(−ΔCt)^ method [23]. The Shapiro-Wilk test was used to assess the normality of variables; as the expression levels (2^(−ΔCt)^) of all analyzed miRNAs deviated from normal distribution, differences between smokers and non-smokers were analyzed using the Mann-Whitney U test. Additionally, the intraclass correlation coefficients (ICC) with a 95% confidence interval (CI) were calculated based on a mean-rating, two-way mixed-effects model, to assess absolute agreement among five different normalizers for each target miRNA. Based on ICC values, the level of agreement was interpreted as follows: poor (<0.5), moderate (0.5–0.75), good (0.75–0.9), or excellent (>0.9) [24].

## Results

### Reference Gene Stability

The overall Ct values obtained by RT-qPCR of five reference genes, along with descriptive statistics obtained using the BestKeeper method are shown in Table 1. All reference genes exhibited Ct values above 21. The largest expression level was observed for miR-143 (mean Ct = 21.40), whereas SNORD48 was the least expressed (mean Ct = 25.77). Regarding expression stability, miR-525 and miR-520c demonstrated the lowest standard deviation (SD) values of 0.871 and 0.949, respectively. On the other hand, miR-135b and miR-143 exceeded the commonly accepted threshold for reliable normalizers (SD < 1) [25]. The SNORD48 (SD(Ct) = 1.048) only slightly surpassed this heuristic threshold and was not excluded from further analyses before estimating its overall stability with other statistical algorithms (GeNorm, NormFinder, and ΔCt method). The BestKeeper index (BKI) was initially calculated as the geometric mean of Ct values of all five candidate reference genes (BKI5), and then recalculated after excluding miR-135b and miR-143 (BKI3) due to their SD values greater than 1. Table 2 shows the results of correlation analysis between individual reference genes, as well as between genes and the calculated BKI values. A strong correlation was observed between miR-520c and miR-143 (r = 0.824, p < 0.001), and between miR-525 and other reference genes (r = 0.787–0.906, p < 0.01), except for miR-135b (r = 0.696, p < 0.001). A moderate to strong correlation (r = 0.627–0.720, p < 0.001) was also found among the other reference genes (miR-520c, miR-135b, and miR-143). Additionally, all reference genes showed high correlations with BKI 5, with miR-525 showing the highest correlation coefficient (r = 0.946, p < 0.01) and miR-135b the lowest one (r = 0.831, p < 0.001). A similar pattern was observed for BKI 3, with the lowest correlation coefficient for miR-135b (r = 0.718, p < 0.001), and the highest for miR-525 (r = 0.962, p < 0.01). Based on the variability of the method, reflected by SD values, and the obtained correlation coefficients, the BestKeeper method identified miR-525 as the most stable reference gene (SD = 0.871; r (BKI 5) = 0.946), followed by miR-520c (SD = 0.949; r (BKI 5) = 0.918) and SNORD48 (SD = 1.048; r (BKI 5) = 0.847).

**TABLE 1:** Descriptive statistics of cycle threshold (Ct) values and the BestKeeper Index (BKI) for five candidate reference genes used to assess gene stability in placental samples using the BestKeeper method.

Reference gene	miR-525	miR-135b	miR-520c	miR-143	SNORD48	BKI 5 (n = 5)	BKI 3 (n = 3)
n	64	64	64	64	64	64	64
Geo. Mean (Ct)	21.65	24.45	24.32	21.37	25.75	23.44	23.84
Ar. Mean (Ct)	21.67	24.47	24.34	21.40	25.77	23.47	23.86
Min (Ct)	19.93	21.98	22.71	18.73	23.59	21.31	22.02
Max (Ct)	23.55	27.26	26.62	24.89	28.00	26.01	25.99
SD (±Ct)	0.871	1.127	0.949	1.296	1.048	1.048	0.953
CV (%)	4.02	4.61	3.90	6.07	4.07	4.47	3.99

SD, Standard deviation; CV, Coefficient of variation; BKI, BestKeeper Index.

**TABLE 2:** Pearson’s linear correlation coefficients (r) between cycle threshold (Ct) values of reference genes and BKI obtained by the BestKeeper method.

Reference gene	miR-525	miR-135b	miR-520c	miR-143	SNORD48
miR-135b	0.696				
miR-520c	0.906	0.687			
miR-143	0.860	0.700	0.824		
SNORD48	0.787	0.627	0.720	0.705	
BKI 5	0.946	0.831	0.918	0.929	0.847
BKI 3	0.962	0.718	0.937	0.854	0.898

BKI 5 – include 5 reference genes; BKI 3 – include 3 reference genes.

Results of reference gene expression stability obtained by the GeNorm and NormFinder methods are shown in Table 3. In the GeNorm analysis, gene stability is assessed based on M-value, where the most stable gene expression is indicated by a lower M-value, and the least stable gene is indicated by a higher M-value. According to this method, a combination of miR-525 and miR-520c demonstrated the highest stability (M-value = 0.401), followed by SNORD48 (M-value = 0.601), while miR-135b was the least stable reference gene, exhibiting the highest M-value (M-value = 0.778) (Table 3). Similarly, the NormFinder algorithm, which ranks genes based on their stability value (S-value), identified miR-525 and miR-520c as the most stable reference genes, with S-values of 0.250 and 0.374, respectively, while miR-135b showed the highest S-value (0.732), indicating its lowest stability among tested genes. The stability rankings obtained by GeNorm and NormFinder were consistent, both identifying miR-525 and miR-520c as the most stable reference genes and miR-135b as the least stable.

**TABLE 3:** Results of gene stability of the tested reference genes in placental samples obtained by the GeNorm and NormFinder methods.

Reference gene	GeNorm	NormFinder
	M-value	S-value
miR-525/miR-520c	0.401	—
miR-525	—	0.250
miR-520c	—	0.374
SNORD48	0.601	0.624
miR-143	0.696	0.645
miR-135b	0.778	0.732

**FIGURE 1:**
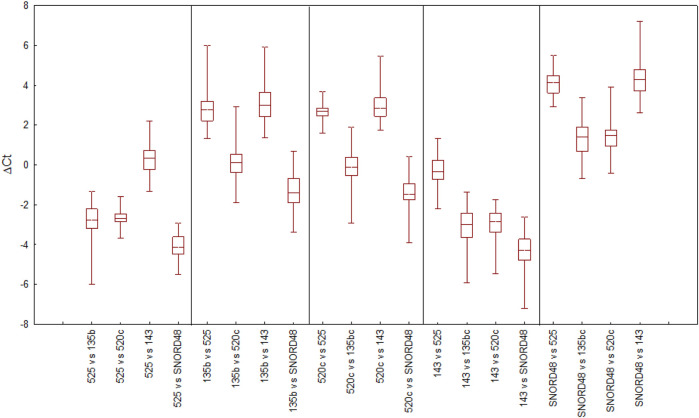
Selection of reference genes in placental samples by the delta Ct method (ΔCt) approach. The variability in ΔCt values for each candidate reference gene is presented as the median (horizontal line), interquartile range (25th -75th percentile; box boundaries), and minimum and maximum values (whiskers).

**FIGURE 2:**
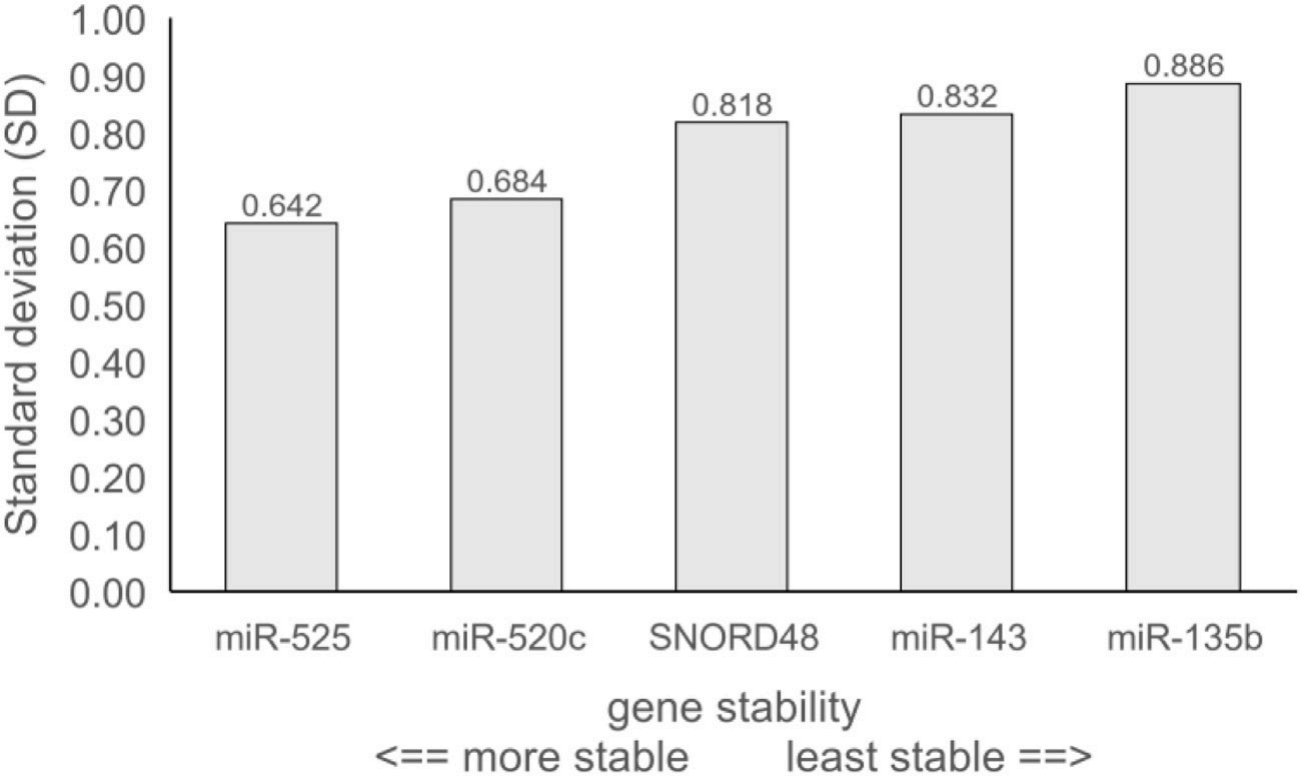
Gene stability of tested reference genes obtained by the delta Ct method (ΔCt). Data are presented as average standard deviation (SD).

The stability of reference gene expression was evaluated by the delta Ct method (ΔCt), in which the mean SD serves as a measure of gene stability. Figure 1 provides an overview of all pairwise gene combinations in placental samples, while Figure 2 shows the average SD for each reference gene across 64 placental samples, where the lower SD value indicates more stable gene expression. All tested genes exhibited an average SD < 1, indicating acceptable gene stability [25]. Notably, miR-525 and miR-520c demonstrated the highest stability in comparison to other reference genes, with SDs of 0.642 and 0.684, respectively. At the same time, miR-135b displayed the lowest level of stability (average SD = 0.886), while SNORD48 and miR-143 showed intermediate stability, with average SDs of 0.818 and 0.832, respectively. Based on the ΔCt method, miR-525 and miR-520c were identified as the most stable reference genes, followed by SNORD48, whereas miR-135b was the least stable.

The overall ranking of gene stability, based on four used statistical approaches, is summarized in Table 4. SNORD48 was identified as a reliable reference gene for normalization across all applied methods, although miR-525, miR-520c, and any combination of these two showed better stability. Across all analyses, miR-135b and miR-143 were consistently ranked as the least stable reference genes, demonstrating the poorest performance.

**TABLE 4:** Overall stability and ranking of reference genes as assessed using different statistical approaches.

Method	Ranking order (better-good-average)				
	1	2	3	4	5
Delta Ct	525	520c	SNORD48	143	135b
BestKeeper	525	520c	SNORD48	135b	143
NormFinder	525	520c	SNORD48	143	135b
GeNorm	525/520c		SNORD48	143	135b

### Effect of Normalizer Selection on Relative Expression Levels

**FIGURE 3:**
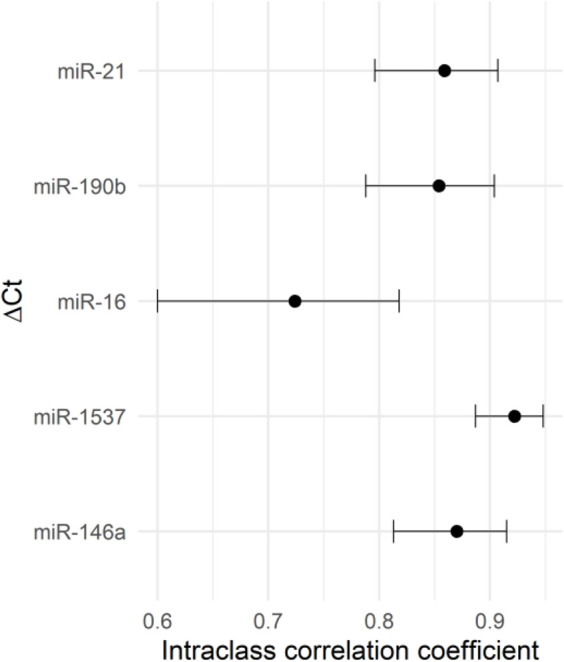
Intraclass correlation coefficient (ICC) with 95% confidence intervals (CI) between RT-qPCR miRNA expression levels of interest, as influenced by different normalizer selection.

**FIGURE 4:**
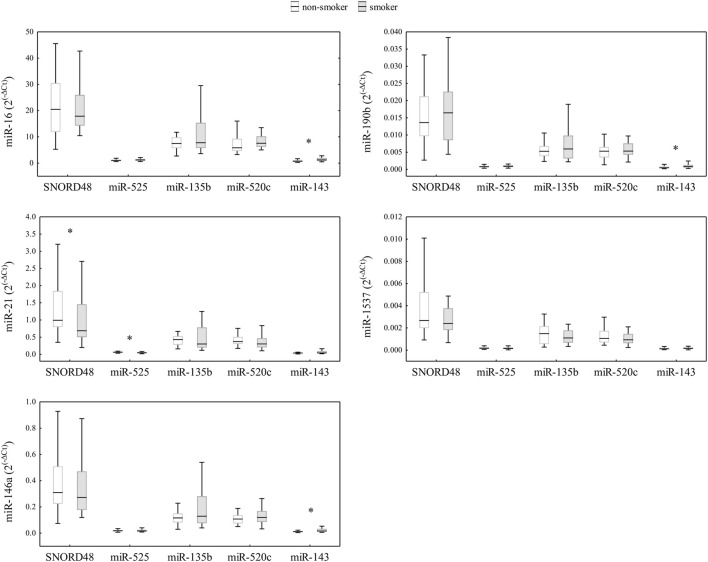
Comparison of miRNA relative expressions (2^(−ΔCt)^) of miR-16, miR-21, miR-146a, miR-190b, and miR-1537 in placenta samples of non-smokers (n = 29) and smokers (n = 35) from Croatia (total n = 64), normalized by various reference genes. Data are presented as median (horizontal line), 25th -75th percentiles (box boundaries), and minimum and maximum (whiskers). Differences between smokers and non-smokers were tested using the Mann-Whitney U-test (p < 0.05), with significant differences indicated by an asterisk.

After assessing the expression stability of reference genes, we investigated the impact of different normalization genes on the quantification of relative expression of five miRNAs of interest (miR-1537, miR-190b, miR-16, miR-21, and miR-146a) in human term placental samples. To begin, we evaluated the intraclass correlation coefficient (ICC) as a measure of absolute agreement between five different reference genes/normalizers (Figure 3). The ICC analysis revealed excellent agreement for miR-1537 (ICC >0.9), and moderate agreement for miR-21, miR-190b and miR-146a. Good, although borderline moderate agreement was found for miR-16 (ICC = 0.724; confidence interval (CI) = 0.600–0.818), indicating that even minor changes in its normalization could affect the interpretation of relative expression results. Furthermore, we performed a sensitivity analysis excluding all imputed observations for miR-1537. The ICC obtained after exclusion was (0.923; CI: 0.883–0.953), which is essentially identical to the ICC obtained with the imputed dataset (0.922; CI: 0.887–0.948). We then analysed the relative expression of candidate miRNAs in placental samples normalized by various reference genes and examined the differences between smokers and non-smokers. To evaluate the miRNA expression differences between smokers and non-smokers, obtained RT-qPCR data were normalized using SNORD48, miR-525, miR-520c, miR-135b, and miR-143. The relative expressions of miRNAs in placental samples, normalized by different normalizers, are shown in Figure 4, and a similar concordance is observed for miR-1537 after removing the imputed data (data not shown). The observed range differences of expression values between different normalizers reflect their endogenous abundance. Our results indicated that normalization by SNORD48 and miR-525 gave similar results, indicating lower expression levels of miR-21 in smokers compared to non-smokers. However, no significant differences were observed when miR-520c was used as a normalizer, although the p-value was close to the significance threshold (smokers: 0.303 (0.104–0.835) vs. non-smokers: 0.372 (0.172–0.756); p = 0.097). Significant differences in miR-16, miR-146a, and miR-190b expression were observed between smokers and non-smokers, with smokers displaying higher levels of these miRNAs when qPCR data were normalized using miR-143.

## Discussion

The RT-qPCR analysis is considered the gold standard for the determination of miRNA expression. The normalization process is an essential step in ensuring the accuracy and reliable quantification of RT-qPCR data [5]. Validation of reference genes used as normalizers is particularly important in miRNA RT-qPCR studies, as miRNAs regulate several target genes, meaning that even little fluctuations in their expression levels may have significant biological and clinical implications. The purpose of normalization is to eliminate variations caused by inconsistencies in sampling, the quantity and quality of the extracted sample, or reverse transcription efficiency. This means that completely abandoning the normalization step [26] or normalizing based solely on the amount of input miRNA [27] and serum volume [28] is not advisable. For example, normalizing gene expression to nanograms of input miRNA ensures that equal quantities of miRNA are compared, but fails to account for potential differences in reverse transcription efficiency. That is why reference genes are commonly employed for normalization. Their use helps to mitigate the variation in reverse transcription efficiency, since both the target miRNA and reference miRNA are reverse-transcribed together [29]. Unlike to the normalization of circulating miRNAs derived from serum/plasma samples, where a synthetic miRNA (i.e. *Caenorhabditis elegans* cel-miR-39) is commonly added as spike-in control during the isolation step and subsequently processed alongside endogenous miRNAs, the normalization of miRNAs in tissue samples requires a fundamentally different approach. This is primarily because miRNA expression levels in tissues are substantially higher compared to those in body fluids, making spike-in controls less effective for correcting technical variation. Instead, normalization in tissue-based studies typically relies on the selection of stable endogenous reference genes to ensure accurate quantification of target miRNAs. Ideally, the chosen reference miRNA should be constantly and consistently expressed across all samples, allowing it to serve as a reliable normalizer that prevents data misinterpretation and enables the detection of actual changes in miRNA expression levels. In this study, we evaluated the stability of five candidate reference genes in human placental tissue samples collected from healthy volunteers after vaginal delivery. The GeNorm and NormFinder algorithms are among the most widely used tools for assessing reference gene stability; however, despite their widespread use, these methods can sometimes lead to suboptimal choices. To ensure a comprehensive evaluation, we also included BestKeeper, and ΔC_t_ method alongside GeNorm and NormFinder. By integrating the results from these four complementary statistical approaches, we systematically evaluated the performance of five candidate reference genes. This multi-method strategy accounted for different aspects of gene stability, including overall and/or intra-group variability, as well as the correlation of expression patterns. To rank the top three reference genes for normalization in placental tissue samples, the most suitable reference genes for accurate normalization were selected by integrating data from all applied algorithms. Additionally, we compared the miRNA expression levels in the placenta from smokers and non-smokers using different normalizers, demonstrating how the selection of normalizer can impact the relative expression of miRNAs and the accurate measure of biological variation.

All statistical methods used in this study consistently identified SNORD48 as one of the good, stable normalizers, reinforcing its widespread use in miRNA normalization. Across multiple algorithms, miR-525 and miR-520c demonstrated even greater stability, suggesting their suitability for normalization in placental tissue, either alone or in combination. However, caution is needed when using them as reference genes, as they have been associated with certain pregnancy-related disorders [30, 31]. Additionally, their role in tumorigenesis—whether as tumor suppressors or promoters—is still under investigation [32–34]. In our dataset, miR-525 and miR-520c showed consistent expression across all placental samples; although this supports their use as normalizers in the present experimental setting, it does not fully exclude their potential involvement in pathophysiology. Accordingly, their potential biological functions restrict their suitability as general-purpose reference genes. Additionally, it is important to understand that there is no single “best” gene for normalization—the stability of reference genes needs to be verified in each specific biological and experimental context. For this reason, SNORD48 is a better choice for normalization, even though miR-520c and miR-525 showed somewhat higher stability ratings. SNORD48, a small nucleolar RNA with no known involvement in placental or pathophysiological processes, can be considered biologically inert, offering a more reliable choice for general normalization than miRNAs with potential regulatory functions. This cautious choice minimizes the risk of confounding effects from reference genes that might respond to pathogenic or physiological stressors. Conversely, miR-135b and miR-143 were consistently ranked as the least stable reference genes, exhibiting highest M-values and variability across all methods, indicating their unsuitability for normalization in human placental samples.

Additionally, we calculated ICC to examine the agreement between different normalizers in quantifying the expression of miRNAs of interest within our population-based cohort of smokers and non-smokers. According to ICC results, excellent or moderate agreement was observed for all candidate miRNAs, with the exception of miR-16, for which agreement ranged from good to borderline moderate. This indicates that slight modifications in the normalization process for miR-16 may lead to variances in the computed relative expression levels, especially in studies with small sample size. Our further analysis revealed that the inferred biological findings can be significantly affected by the choice of normalizer. Normalization by SNORD48 and miR-525 gave concordant results, revealing a significant difference in placental miR-21 relative expression between smokers and non-smokers. In contrast, normalization with miR-520c did not reach statistical significance (p = 0.097), despite miR-520c being ranked among the top three reference genes in our stability analysis. This divergence highlights that high stability rankings do not necessarily translate into biological usefulness. Likely contributors to this discrepancy include small sample size, biological variability between samples, and deviations in baseline expression levels. Accordingly, to detect physiologically meaningful changes, reference gene selection should balance practical, biological relevance with technical factors. Beyond methodological considerations, the findings also have biological significance, as miRNA downregulation in placental tissue may impact cell proliferation and migration [35]. Specifically, miR-21 is a well-described regulator of processes related to proliferation, migration, and response to oxidative/inflammatory stress [35, 36], which are all relevant to smoking-related placental alterations [14]. In this light, the reduced expression of miR-21 in the placenta of smokers compared to non-smokers (when normalized by SNORD48 and miR-525) is biologically plausible and aligns with the findings of Maccani et al., [14] who reported downregulation of miR-21, miR-16, and miR-146a in placentas of smokers. Although our study did not detect statistically significant differences for miR-16 and miR-146a, the results indicate a trend toward lower expression of these miRNAs in smokers. On the other hand, normalization using miR-143 as a reference gene produced results that differed considerably from those obtained with more stable miRNA controls (SNORD48 and miR-525). Specifically, several miRNAs of interest–including miR-16, miR-190b, and miR-146a–exhibited statistically significant upregulation in placental tissue of smokers when normalized with miR-143. These results should be interpreted with caution, as the observed higher expression of miR-16 and miR-146a in smokers compared to non-smokers contradicts both the trends reported in previous studies Maccani et al., [14] and Li et al., [13] and our own results obtained using empirically validated stable genes, which consistently showed lower expression of these miRNAs in smokers. Given that miR-143 demonstrated the poorest stability across experimental conditions in our reference gene analysis, we attribute these discrepancies to normalization bias rather than true biological variation. Collectively, these results show how even small variations in reference gene stability can significantly affect data interpretation. Therefore, instead of relying on a single universally accepted normalizer, reference gene selection should be based on empirical evaluation performed in the same tissue type and experimental context as the samples under investigation. In clinical research, where gene expression findings can directly impact diagnostic and therapeutic decisions, use of an inappropriate reference gene could lead to misleading conclusions and potentially incorrect diagnosis [37].

### Limitations

This study has several weaknesses. First, the selection of candidate reference genes was based on manufacturer recommendations and/or literature evidence–specifically, among miRNAs known to be well expressed in placental tissue or previously used as housekeeping genes–explicitly excluding from consideration the miRNAs that were the primary subjects of this study. This pragmatic strategy may have inadvertently excluded other suitable and potentially more stable small RNAs (e.g. U6 and RNU6B) or uncharacterized candidates that might have been identified with a broader screening panel of reliable reference genes. In principle, although genome-wide approaches like RNA sequencing or microarray analysis could have been used to discover additional novel candidate reference genes, the implementation of such methods was beyond the purview and budget of the current study. Second, our research was restricted to placental tissue only and relied on a single sample of placental trophoblastic tissue per participant. Given the well-known heterogeneity of placental tissue, which may influence its transcriptomic profile [38], this sampling strategy is an important source of measurement uncertainty. To partially mitigate this, all samples were taken from the same predefined location of the placental disc. Nevertheless, future studies should, if feasible, include multiple samples from different regions of the same placenta to minimize overall variability and provide a more representative expression profile. Third, in instances when Ct values were undetermined or exceeded 35.4 (for miR-1537), we applied a single imputation of the data by assigning Ct value of 36 (Ct + 1). While this approach may have introduced some degree of bias in the final estimations, outright deletion of these observations would have further reduced the effective sample size and statistical power, potentially limiting our ability to detect meaningful differences. We therefore prioritized retention of all biological replicates, noting that results for miR-1537 should be interpreted with appropriate caution. Fourth, since all analyses were conducted on a single set of placental samples, the observed stability of reference miRNAs (miR-525, miR-520c, and SNORD48) should be further validated in independent cohorts to assess robustness and generalizability. Notwithstanding this limitation, our findings provide useful foundation for selection of stable reference genes in placental miRNA research and a solid basis for future research in other populations and different experimental conditions.

In conclusion, our findings highlight the critical importance of selecting and empirically validating reference genes within the specific experimental and clinical setting in which they will be used. Although miR-520c and miR-525 showed overall good stability in our placental samples, their use as normalizers should be interpreted cautiously, as their potential involvement in placental or pregnancy-related pathophysiology cannot be entirely excluded. Conversely, SNORD48 was stable in our dataset and, to our knowledge, has no known role in pathophysiology, making it a more reliable choice for normalization since it reduces the risk of confounding by the biological activity of the reference gene itself. Overall, these results highlight that context-appropriate reference gene selection is essential for improving the accuracy and reliability of RT-qPCR-based analyses in placental and other tissues, rather than looking for a single “correct” or universal normalizer. Additionally, it is essential to remember that the stability of reference genes is heavily context dependent and can be influenced by many factors, including which samples are included or excluded, differences between cohorts, variations in the spatial origin of placental biopsies, and potential inclusion of pathological placentas. These methodological and biological sources of variability should be carefully considered when comparing data across studies or when interpreting normalization-dependent outcomes.

## Summary Table

### What Is Known About This Subject

- Normalization is a crucial step in RT-qPCR analysis.
- Reference genes validated in one tissue type might not be suitable for another.
- Evidence on suitable reference miRNAs in placental tissue is currently limited.

### What This Paper Adds

- miR-520c and miR-525 show stable expression in placental samples, but caution is needed due to their possible biological involvement.
- SNORD48 is stable and has no known pathophysiological role, making it a reliable normalizer.
- Using less stable reference genes, such as miR-143, may produce results that differ more significantly from those obtained with stable miRNA controls.

### Concluding Statement

This work represents an advance in biomedical science by demonstrating that selecting the appropriate reference gene is crucial for enhancing the accuracy and reliability of RT-qPCR-based analyses in placental and other tissues.

## Data Availability

The data that support the findings of this study are available from the corresponding author, upon reasonable request.

## References

[1] Bartel DP . MicroRNAs: Genomics, Biogenesis, Mechanism, and Function. Cell (2004) 116(2):281–97. 10.1016/s0092-8674(04)00045-5 14744438

[2] Livak KJ Schmittgen TD . Analysis of Relative Gene Expression Data Using Real-Time Quantitative PCR and the 2(-Delta Delta C(T)) Method. Methods (2001) 25(4):402–8. 10.1006/meth.2001.1262 11846609

[3] Rai S Ray Y Pan H Prabhu S Hamid T . Statistical Analysis of Repeated MicroRNA High-Throughput Data with Application to Human Heart Failure: A Review of Methodology. Open Access Med Stat (2012) 2012(2):21–31. 10.2147/OAMS.S27907 24738042 PMC3984897

[4] Bustin SA Beaulieu JF Huggett J Jaggi R Kibenge FSB Olsvik PA MIQE Précis: Practical Implementation of Minimum Standard Guidelines for Fluorescence-Based Quantitative Real-Time PCR Experiments. BMC Mol Biol (2010) 11:74. 10.1186/1471-2199-11-74 20858237 PMC2955025

[5] Schwarzenbach H Da Silva AM Calin G Pantel K . Data Normalization Strategies for MicroRNA Quantification. Clin Chem (2015) 61(11):1333–42. 10.1373/clinchem.2015.239459 26408530 PMC4890630

[6] Spiekermann M Dieckmann KP Balks T Bullerdiek J Belge G . Is Relative Quantification Dispensable for the Measurement of Micrornas as Serum Biomarkers in Germ Cell Tumors? Anticancer Res (2015) 35(1):117–21. 25550541

[7] Wang X Gardiner EJ Cairns MJ . Optimal Consistency in MicroRNA Expression Analysis Using Reference-Gene-Based Normalization. Mol Biosyst (2015) 11(5):1235–40. 10.1039/c4mb00711e 25797570

[8] Kozera B Rapacz M . Reference Genes in Real-Time PCR. J Appl Genet (2013) 54(4):391–406. 10.1007/s13353-013-0173-x 24078518 PMC3825189

[9] Dheda K Huggett JF Chang JS Kim LU Bustin SA Johnson MA The Implications of Using an Inappropriate Reference Gene for Real-Time Reverse Transcription PCR Data Normalization. Anal Biochem (2005) 344(1):141–3. 10.1016/j.ab.2005.05.022 16054107

[10] Noguer-Dance M Abu-Amero S Al-Khtib M Lefèvre A Coullin P Moore GE The Primate-Specific MicroRNA Gene Cluster (C19MC) Is Imprinted in the Placenta. Hum Mol Genet (2010) 19(18):3566–82. 10.1093/hmg/ddq272 20610438

[11] Barad O Meiri E Avniel A Aharonov R Barzilai A Bentwich I MicroRNA Expression Detected by Oligonucleotide Microarrays: System Establishment and Expression Profiling in Human Tissues. Genome Res (2004) 14(12):2486–94. 10.1101/gr.2845604 15574827 PMC534673

[12] Enquobahrie DA Abetew DF Sorensen TK Willoughby D Chidambaram K Williams MA . Placental MicroRNA Expression in Pregnancies Complicated by Preeclampsia. Am J Obstet Gynecol (2011) 204(2):178.e12–21. 10.1016/j.ajog.2010.09.004 21093846 PMC3040986

[13] Li Q Kappil MA Li A Dassanayake PS Darrah TH Friedman AE Exploring the Associations Between microRNA Expression Profiles and Environmental Pollutants in Human Placenta from the National Children’s Study (NCS). Epigenetics (2015) 10(9):793–802. 10.1080/15592294.2015.1066960 26252056 PMC4622837

[14] Maccani MA Avissar-Whiting M Banister CE McGonnigal B Padbury JF Marsit CJ . Maternal Cigarette Smoking During Pregnancy Is Associated with Downregulation of miR-16, miR-21, and miR-146a in the Placenta. Epigenetics (2010) 5(7):583–9. 10.4161/epi.5.7.12762 20647767 PMC2974801

[15] Sekovanić A Jurasović J Piasek M Pašalić D Orct T Grgec AS Metallothionein 2A Gene Polymorphism and Trace Elements in Mother-Newborn Pairs in the Croatian Population. J Trace Elem Med Biol (2018) 45(2018):163–70. 10.1016/j.jtemb.2017.10.011 29173474

[16] Piasek M Škrgatić L Sulimanec A Orct T Sekovanić A Kovačić J Effects of Maternal Cigarette Smoking on Trace Element Levels and Steroidogenesis in the Maternal–Placental–Fetal Unit. Toxics (2023) 11:714. 10.3390/toxics11080714 37624219 PMC10459679

[17] Sekovanić A Dorotić A Jurasović J Pašalić D Kovačić J Stasenko S Pre-Amplification as a Method for Improvement of Quantitative RT-PCR Analysis of Circulating miRNAs. Biochem Med (2021) 31(1):010901. 10.11613/BM.2021.010901 33380895 PMC7745165

[18] Gevaert AB Witvrouwen I Vrints CJ Heidbuchel H Van Craenenbroeck EM Van Laere SJ MicroRNA Profiling in Plasma Samples Using qPCR Arrays: Recommendations for Correct Analysis and Interpretation. PLoS One (2018) 13(2):e0193173–13. 10.1371/journal.pone.0193173 29474497 PMC5825041

[19] Pfaffl MW Tichopad A Prgomet C Neuvians TP . Determination of Stable Housekeeping Genes, Differentially Regulated Target Genes and Sample Integrity: Bestkeeper - Excel-Based Tool Using Pair-Wise Correlations. Biotechnol Lett (2004) 26(6):509–15. 10.1023/b:bile.0000019559.84305.47 15127793

[20] Vandesompele J De Preter K Pattyn F Poppe B Van Roy N De Paepe A Accurate Normalization of Real-Time Quantitative RT-PCR Data by Geometric Averaging of Multiple Internal Control Genes. Genome Biol (2002) 3(7):RESEARCH0034–12. 10.1186/gb-2002-3-7-research0034 12184808 PMC126239

[21] Andersen CL Jensen JL Ørntoft TF . Normalization of Real-Time Quantitative Reverse transcription-PCR Data: A Model-Based Variance Estimation Approach to Identify Genes Suited for Normalization, Applied to Bladder and Colon Cancer Data Sets. Cancer Res (2004) 64(15):5245–50. 10.1158/0008-5472.CAN-04-0496 15289330

[22] Silver N Best S Jiang J Thein SL . Selection of Housekeeping Genes for Gene Expression Studies in Human Reticulocytes Using Real-Time PCR. BMC Mol Biol (2006) 7:33–9. 10.1186/1471-2199-7-33 17026756 PMC1609175

[23] Pfaffl MW . A New Mathematical Model for Relative Quantification in Real-Time RT–PCR. Nucleic Acids Res (2001) 29(9):e45. 10.1093/nar/29.9.e45 11328886 PMC55695

[24] Koo TK Li MY . A Guideline of Selecting and Reporting Intraclass Correlation Coefficients for Reliability Research. J Chiropr Med (2016) 15(2):155–63. 10.1016/j.jcm.2016.02.012 27330520 PMC4913118

[25] Masè M Grasso M Avogaro L D’Amato E Tessarolo F Graffigna A Selection of Reference Genes Is Critical for miRNA Expression Analysis in Human Cardiac Tissue. A Focus on Atrial Fibrillation. Sci Rep (2017) 7(January):41127. 10.1038/srep41127 28117343 PMC5259703

[26] Yan W Li R Liu Y Yang P Wang Z Zhang C MicroRNA Expression Patterns in the Malignant Progression of Gliomas and a 5-MicroRNA Signature for Prognosis. Oncotarget (2014) 5(24):12908–15. 10.18632/oncotarget.2679 25415048 PMC4350338

[27] Yau TO Wu CW Dong Y Tang CM Ng SSM Chan FKL MicroRNA-221 and MicroRNA-18a Identification in Stool as Potential Biomarkers for the Non-Invasive Diagnosis of Colorectal Carcinoma. Br J Cancer (2014) 111(9):1765–71. 10.1038/bjc.2014.484 25233396 PMC4453736

[28] Wang J Pei Y Zhong Y Jiang S Shao J Gong J . Altered Serum MicroRNAs as Novel Diagnostic Biomarkers for Atypical Coronary Artery Disease. PLoS One (2014) 9(9):e107012. 10.1371/journal.pone.0107012 25198728 PMC4157840

[29] Bio-Rad L . Bio-Rad Protocol Guide. BioRadiations (2007) 121.

[30] Liang L Chen Y Wu C Cao Z Xia L Meng J MicroRNAs: Key Regulators of the Trophoblast Function in Pregnancy Disorders. J Assist Reprod Genet (2023) 40(1):3–17. 10.1007/s10815-022-02677-9 36508034 PMC9742672

[31] Mohamad MA Manzor NFM Suhiman MS Sathar J Rahman HA Masri M MicroRNA and Gene Expression Analysis on Placenta Tissue: An Approach to Understanding Obstetric Antiphospholipid Syndrome at the Molecular Level. bioRxiv (2019):780114. 10.1101/780114

[32] Chen Y Gao B Pan Y Wang Q Zhang Q . MiR-525-5p Modulates Cell Proliferation, Cell Cycle, and Apoptosis in Burkitt’s Lymphoma by Targeting MyD88 and Regulating the NF-κB Signaling Pathway. Ann Hematol (2024) 103:5817–33. 10.1007/s00277-024-06062-7 39495280

[33] Huang Q Gumireddy K Schrier M le Sage C Nagel R Nair S The microRNAs miR-373 and miR-520c Promote Tumour Invasion and Metastasis. Nat Cell Biol (2008) 10(2):202–10. 10.1038/ncb1681 18193036

[34] Azimzadeh-Isfanjani A Safaralizadeh R Hosseinpour-Feizi M Shokouhi B Nemati M Moaddab SY . Expression of miR-520c in Intestinal Type Gastric Adenocarcinoma. J Gastrointest Oncol (2018) 9(6):1184–9. 10.21037/jgo.2018.08.09 30603140 PMC6286939

[35] Addo KA Palakodety N Hartwell HJ Tingare A Fry RC . Placental MicroRNAs: Responders to Environmental Chemicals and Mediators of Pathophysiology of the Human Placenta. Toxicol Rep (2020) 7:1046–56. 10.1016/j.toxrep.2020.08.002 32913718 PMC7472806

[36] Kumarswamy R Volkmann I Thum T . Regulation and Function of miRNA-21 in Health and Disease. RNA Biol (2011) 8(5):706–13. 10.4161/rna.8.5.16154 21712654 PMC3256347

[37] Faraldi M Gomarasca M Banfi G Lombardi G . Free Circulating miRNAs Measurement in Clinical Settings: The Still Unsolved Issue of the Normalization. Adv Clin Chem (2018) 87:113–39. 10.1016/bs.acc.2018.07.003 30342709 PMC7112021

[38] Xie L Mouillet JF Chu T Parks WT Sadovsky E Knöfler M C19MC MicroRNAs Regulate the Migration of Human Trophoblasts. Endocrinology (2014) 155(12):4975–85. 10.1210/en.2014-1501 25211593 PMC4239420

